# Community-based housing alternatives for older adults: towards a conceptual framework for resident involvement

**DOI:** 10.1007/s10433-025-00895-5

**Published:** 2025-12-03

**Authors:** Luise Stoisser, Tine Buffel, Ann Petermans, An-Sofie Smetcoren

**Affiliations:** 1 Society and Ageing Research Lab (SARLab), Vrije Universiteit Brussels (VUB), Brussels, Belgium; 2 Department of Sociology, the Manchester Urban Ageing Research Group (MUARG), The University of Manchester, Manchester, England; 3 Faculty of Architecture and Arts, Research Group ARCK—Designing for More, Hasselt University, Hasselt, Belgium; 4 Society and Ageing Research Lab (SARLab), Vrije Universiteit Brussels (VUB), Brussels, Belgium

**Keywords:** Ageing in place, Community-based housing, Co-production, Governance

## Abstract

Most older adults in Europe want to age in their own homes. However, this is not feasible or desirable for everyone. Limited financial resources, lack of daily support or social contact, the sudden loss of a partner, or a desire for change may result in a wish or necessity to move. Community-based housing alternatives, such as co-housing, naturally occurring retirement communities (NORCs), sheltered housing, or villages, can provide viable options. These initiatives facilitate independent living, care and support, and a sense of community. Compared to institutionalised care homes, community-based housing offers autonomy, independent living, and the opportunity to shape one’s home environment. However, despite variation in how residents participate in co-producing their living environments, the role of resident involvement in shaping community-based housing has been underexplored. To address this gap, this paper proposes a conceptual framework for understanding how older residents engage in co-producing community-based housing. Bringing together literature on housing co-production and community-based housing for older adults, the framework distinguishes between community-*led* and community-*oriented* co-production. Community-*led* co-production refers to practices that are controlled by older residents, while community-*oriented* co-production describes practices that include resident input, but led by other stakeholders. By developing and discussing this framework, the paper lays the groundwork for future empirical studies and offers guidance for policymakers, practitioners, and housing providers on considering the role of residents in shaping future housing models for older adults.

## Introduction

*Ageing in place* refers to both a policy goal and a commonly expressed aspiration among older adults to remain in their homes and communities as they grow older (Pani-Harreman et al. 2021). This is often linked to strong attachments to place and a desire to maintain autonomy, especially as ageing-related physical, cognitive, and social changes increase reliance on the immediate environment (Lebrusán and Gómez 2022; Tournier and Vidovićová 2021). However, not everyone can or wants to age in place (Hillcoat-Nallétamby and Ogg 2014). Some lack informal support or experience loneliness (Barrett et al. 2012), while others face financial insecurity in housing markets shaped by crises (Potts 2020). Institutional care homes^1^ are not always seen as viable options, as they are often perceived as restricting older residents’ autonomy and control over daily life (Gould et al. 2017; Fernández-Carro 2016). In this context, community-based housing developments have emerged as a promising alternative. We define community-based housing developments as housing arrangements that a) provide dwellings for independent living in a defined spatial area, such as parts of a building, a housing block, or parts of a neighbourhood, b) offer some level of care and support, and c) aim to foster a sense of community among residents. As such, these models are designed to support ageing in ways that combine autonomy with social connection (Mahmood et al. 2021; Chum et al. 2022).

Ageing studies have increasingly called for less institutionalised housing solutions, emphasising that incorporating older resident voices and perspectives in governance is essential for ensuring that housing and care meet residents’ needs (Greenfield 2018; Buffel, Yarker, and Doran 2024; Stoisser et al. 2025). One approach to achieving this is through *co-production,* understood here as collaboration between user groups (i.e. in this paper, older residents) and housing providers across a continuum of involvement, ranging from consultation of residents in co-production to resident-led models (Czischke 2018). In community-based housing, co-production may involve participation in the design of buildings and services, the selection of residents, and the ongoing daily management of the housing project. Such arrangements often involve diverse actors, including civil society, market, and state stakeholders. While Czischke’s framework was developed in the context of co-housing—typically a self-organised model of community-based housing—the focus on shared governance between residents and institutions offers broader relevance for understanding how older adults might exercise greater influence over their housing environments.

Importantly, community-based housing projects for older adults vary in how they include residents in governance. Both housing studies and ageing research discuss these developments, revealing a wide range of co-production arrangements. Housing studies typically focus on self-organised housing schemes, such as co-housing—in which residents manage the housing collectively, combining private living spaces with communal facilities and community life (Crabtree-Hayes 2024). Ageing studies, in contrast, often also consider more provider-led schemes, such as sheltered housing, which offers purpose-built accommodation that combines independent living with on-site support and shared social spaces (E.g. Chum et al. 2022). Existing reviews (Chum et al. 2022; Mahmood et al. 2021) and studies (e.g. Power 2017; Rosenberg et al. 2018; Glass 2020) show that these housing options vary significantly in the way they organise independent living, care and support, and sense of community. While some models, such as co-housing, rely on active resident participation in service provision and governance, others, like sheltered housing, are typically managed by private or public housing providers with limited resident involvement in design, development, or daily operation.

Yet, despite this variation, the broader role of residents across different forms of community-based housing remains insufficiently explored. Existing research has largely examined one specific type of community-based housing at a time, typically through single or multiple case studies (E.g. Graham, Scharlach, and Price Wolf 2014; Hammond 2018), with fewer studies offering comparative analyses across different models (Davitt et al. 2017; Hou and Cao 2021). Even fewer papers have sought to develop conceptual tools to analyse the nature and extent of resident involvement across different types of community-based housing for older adults. To address this gap, this paper develops a conceptual framework for understanding resident involvement in community-based housing developments. By conceptualising how older adults can influence the elements of independent living, care and support, and a sense of community, this paper introduces a framework that describes community-based housing as a mix of community-led (controlled by residents) and community-oriented (managed by other stakeholders) governance practices. In doing so, it provides a foundation for further empirical research and supports policymakers, practitioners, and housing providers in reflecting on the role of residents in shaping future housing developments for older adults.

The paper is structured as follows: first, we draw on the literature on different types of community-based housing and examine how these models co-produce independent living, care and support, and a sense of community in later life. We then introduce a framework for understanding resident involvement across these domains. Finally, the paper discusses the implications of this conceptualisation for practitioners and academics, with a particular focus on the benefits and drawbacks of both community-led and community-oriented housing provision for an ageing population.

## Resident involvement across community-based housing types

Contemporary studies reveal how independent living, care and support, and a sense of community are co-produced with older adults across different types of community-based housing. This section reviews literature on four housing types, selected for their distinct co-production arrangements: co-housing, villages, sheltered housing, and naturally occurring retirement communities (NORCs). Co-housing and villages are typically grassroots initiatives with high levels of resident involvement, whereas sheltered housing and NORCs are predominantly provider-led and publicly managed. These models also often vary in spatial scale: co-housing and sheltered housing are usually located within single buildings or housing blocks, while villages and NORCs typically operate on a neighbourhood scale. The literature we draw upon predominantly comes from Northern/Western Europe and the USA, where most English-speaking work on this topic has been conducted. The four types of community-based housing projects can be briefly described as follows:

- Co-housing projects originated in Europe as part of resident-led housing movements, involving developments that are collectively designed, built, and managed by residents who wish to live together in an intentional community while maintaining their own private living spaces (Czischke et al. 2020).
- Villages originated in the USA as ‘grassroots, consumer-driven membership organisations typically developed and governed by older community members’ (Scharlach and Lehning 2013, 119). Through collective organisation of staff and volunteers, they provide services such as health and technology support and social opportunities for members living in a defined area.
- Naturally occurring retirement communities (NORCs) also emerged in the USA. NORCs are existing housing blocks or neighbourhoods not initially meant for older people, but with an unintentionally high proportion of older adults (Greenfield 2016). By establishing so-called supportive service programmes (NORC-SSPs), NORCs connect residents to community and health services in the area (E et al. 2022).
- Sheltered housing offers purpose-built, often affordable, supportive independent living with social opportunities such as shared meals and events. Services offered, such as a warden or professional care staff, and access criteria vary across national contexts (see Corneliusson et al. 2019; Chum et al. 2022).

The next sections examine how each of these housing models aims to provide the three elements of community-based housing—independent living, care and support, and a sense of community.

### Independent living

Community-based housing initiatives for older adults provide a place to age independently. This includes, first and foremost, a dwelling for *independent living*. The physical layouts of dwellings can vary across initiatives and local policy contexts, but generally include everything needed for independent living—kitchen, living room, bathroom(s), and bedroom(s). The dwelling might have adaptable layouts to accommodate changing preferences as one ages and shared spaces to enable social contact (Chum et al. 2022). Crucially, independent living is not just about having the physical space to live independently, but also about having the autonomy to personalise and change one’s living space. This autonomy is often shaped by the project’s governance structure and tenure arrangements, which may include owner-occupation, cooperative ownership, social or private rental, or mixed-tenure models within the same development.

Co-housing, for example, is typically community-led. Residents often decide what spaces they want and what these should look like (Beck 2020). Co-housing can be privately owned, organised through housing cooperatives with shared ownership, private renting, or public housing owned by a non-profit housing association (Beck 2020; see also Bossuyt 2022). Pedersen (2015), in the context of Denmark, demonstrates that senior co-housing can take various tenure forms beyond private ownership, including social rental, private cooperative ownership, and private rental (see also Riccò et al. 2024). Importantly, co-housing projects vary when it comes to the degree of resident involvement (Czischke 2018). The increasing involvement of social housing organisations or city governments in co-housing often comes with rental arrangements instead of private ownership and thus a decrease in resident autonomy (e.g. when designing the spaces or choosing co-residents, Stoisser and van Gent 2025). At the same time, rental tenure and greater involvement of housing providers or municipalities may broaden access by enabling the inclusion of residents from more diverse socioeconomic backgrounds (Droste 2015) and with varying health needs (Pedersen 2015).

Villages, which are purposefully built with older adults in mind, also emphasise independent living with accessible spaces that accommodate care needs. Graham et al. (2018) describe how five villages in California, USA, focus on enabling independent living to prevent relocation into an institutional care facility. Villages function on a broader scale and rely on membership. In most villages in the USA, residents initiate, develop, and continue to influence village management (Graham, Scharlach, and Price Wolf 2014; Hou and Cao 2021). However, not all villages are grassroot-driven. They can be privately run for profit, operated by a private provider and funded by member fees, run by charity organisations, or organised through private–public partnerships (Osei-Kyei et al. 2022). Osei-Kyei et al. (2022) explored the latter through a global survey addressing collaboration between governments and private developers. They found that these collaborations can make villages more affordable compared to private initiatives that often cater to more affluent and white audiences (see also Graham et al. 2018), but also come with challenges, such as a limited understanding of the design needs of older people.

Sheltered housing projects offer living arrangements that ‘foster independent living with a secondary focus on providing affordable accommodations for its residents’ (Chum et al. 2022, 189). In the UK, most sheltered housing is provided by local authorities or housing associations that have specialised in older adult housing (Butler et al. 2021). As in conventional social rental housing, residents in sheltered housing can often bring their furniture and shape its interiors and, because it is designed for older adults, spaces are accessible for different care needs. However, residents are typically less involved in the development and management of projects than in co-housing and villages.

Naturally occurring retirement communities offer a different approach to supporting independent living. These community initiatives develop naturally in housing blocks where a large population of older adults resides. The concept originated in the USA, and most research focuses on the US context (E et al. 2022). There, older adults continue to live in their own rented or owned independent dwellings, which were not originally designed to be accessible for older adults. A NORC structure is then established in this area, as will be explained in the next section on support and care.

### Support and care

To live independently, older adults might not only need an independent dwelling but also accessible support and care. *Support* can range from more passive forms (e.g. through barrier-free design) to active support, such as building maintenance, meals, and organised daily activities (Howe et al. 2013; Hou and Cao 2021). *Care* services are usually provided one-to-one between a care recipient and a caregiver and are commonly related to health care (Howe et al. 2013). Support and care services can be provided informally through neighbours, family, friends, or the broader community or formally through organised and professional care and support providers.

Co-housing models rely primarily on the community’s collective responsibility to provide support in daily life and on informal care services. Puplampu (2020) shows that older adults in co-housing in western Canada care about and for each other—they describe how those in better health drive neighbours to the doctor or cook meals. Co-housing, thus, can offer informal care and support within the housing project, and residents normally have a role in providing this support to each other. Through a literature review and case studies in Sweden, Germany, and England, Labit (2015) finds that solidarity, especially intergenerational solidarity, comes from affinity and is difficult to engineer by design—those who like each other help each other. Co-housing might not be suitable for older adults with high care needs since the community might lack the resources to provide care once residents become more dependent (Labit 2015). However, not all co-housing groups rely only on residents when it comes to organising care, as shown in a study of a project in Brussels. There, the project had initially outsourced the organisation and conceptualisation of care to an NGO. Now, this NGO is no longer involved, and the responsibility for organising care has been fully transferred to the residents (Dawance et al. 2019) following an intentional shift from ‘associative governance’ to ‘inhabitant governance’ (Smetcoren et al. 2022, 85; Mosseray et al. 2025).

Similar to co-housing, but on a neighbourhood scale instead of one housing complex, villages can offer support and care through village members. Hou and Cao (2021) describe that in villages in the USA, services are mostly provided by volunteers. This means that people in a specific area become members and subscribe to receiving or giving care and support, but that support is not provided by professional and paid staff. Like co-housing, the co-production arrangement of care in such villages relies on a high degree of resident involvement. However, not all villages work with resident participation at their core. Others might collaborate with municipalities or private social companies to offer care/support services (e.g. example of ten villages in Finland, Lundman 2020).

Sheltered housing often provides support services, such as a warden, a 24-h alarm system, or activities to keep physically active (e.g. as Herbers and Meijering 2015 observed in the Netherlands). The community in sheltered housing is less interdependent than in co-housing because people do not self-organise to manage the housing project. However, Cook et al. (2017) show that the communal elements of sheltered housing can nonetheless create opportunities for support and solidarity among residents in the UK. Thus, informal and self-organised support through neighbours also emerges within these housing forms (see also Herbers and Meijering 2015), even if it is not planned.

Likewise, but on a larger geographical scale, NORCs provide formalised care and support. They often establish a so-called supportive service programme (NORC-SSP), as Hou and Cao (2021) explain in their review of the US context. This is a programme led by NGOs or private service providers (e.g. Enguidanos et al. 2010 for the US context) that creates collaborations between people ageing in NORC areas, local health providers, and social service providers (E et al. 2022). Services can include transportation assistance, health services, recreation and education programmes, and volunteer opportunities (E et al. 2022). Based on a scoping review of North American cases, Parniak et al. (2022) found that most NORCs collaborate with external organisations such as home care agencies, local hospitals, nursing homes, transportation agencies, or universities. While NORCs are prominent in the USA, few examples exist in Europe, and even fewer have been studied. However, Kavanagh et al. (2025) describe a developing NORC in a high-rise building in the UK, discussing how tenants co-create the NORC and play a crucial role in determining services offered, e.g. an independent living worker who supports older tenants. This NORC is developed with a high level of resident participation in co-production and comes with challenges such as securing long-term funding for the employed worker and establishing a relationship of trust with the housing provider.

### Sense of community

Community-based housing also aims to enable a sense of community among residents. While a functioning community cannot easily be engineered by design, community-based housing projects can support a sense of community, for example, through built spaces that enable interaction and by organising events.

In co-housing groups, this sense of community is intentional, and what the community should entail is part of the co-production of the development (Jarvis 2015a). Typically, members of co-housing pursue a collective life based on shared values and purpose, often forming their community long before moving into the housing project and deciding collectively who will live in the project. Glass (2020) shows that a majority of older adults move into co-housing because of its strong community aspect, with research confirming that senior co-housing promotes self-organised activities and a satisfactory sense of community (see also Glass and Norris 2023). Studying co-housing groups in the UK, USA, and Australia, Jarvis (2015b) found that the social architecture of co-housing is complex but essential for the success of such groups. Interestingly, Jarvis refers to the influence of different types of co-production on the sense of community: arrangements with stakeholders beyond the resident group can improve the quality of interpersonal relationships (for example, through communication training). At the same time, it can also create power asymmetries, leading to complex group dynamics.

In contrast, sheltered housing presents a less intentional community, with residents typically choosing their place for ageing after the project is built, often allocated through social housing systems (see E.g. Fox et al. 2017). Nonetheless, these projects also organise community spaces and activities. Fox and colleagues (2017) found that, in Ireland, sheltered housing residents joined social activities significantly more often than residents of regular social housing, and many sheltered housing residents feel part of their community. Residents of sheltered housing in the Netherlands also benefit from social contact with caregivers and housekeepers, as well as relationships of support with their neighbours (Herbers and Meijering 2015).

Compared to co-housing, where units are often allocated based on collective decision-making, NORCs, sheltered housing, and villages do not typically involve residents in unit allocation. NORCs, with their formalised supportive service programmes, connect residents of NORC areas to community programmes such as social-recreational activities, educational activities, and civic engagement activities to support ageing in place and foster connections (Hou and Cao 2021). Similarly, villages aim to create connections through recreational activities and opportunities for civic participation organised by volunteers or partnering organisations (Graham et al. 2018). In 10 villages in Finland, social activities are organised by members of a non-profit organisation, which is founded for this purpose in each village and can be joined by village residents (Lundman 2020). In this way, residents co-create and shape the community. The initiative for more social connections can also come entirely from residents. In the UK-based NORC described by Kavanagh et al. (2025), tenants have redesigned a ground-floor apartment to be turned into a community space to enable social interaction and combat social isolation faced by many single-dwelling older adults in the building. To achieve this, they collaborate with the housing provider. Further, a sense of community can be shaped by many factors beyond the control of residents. Bernard and colleagues (2012) describe in detail the experiences of sense of community in a village in the UK. Among others, the size of the village, as well as the changes in residents over time, influenced how village members felt a sense of community.

## Towards a framework: resident involvement in community-based housing

**Fig. 1:**
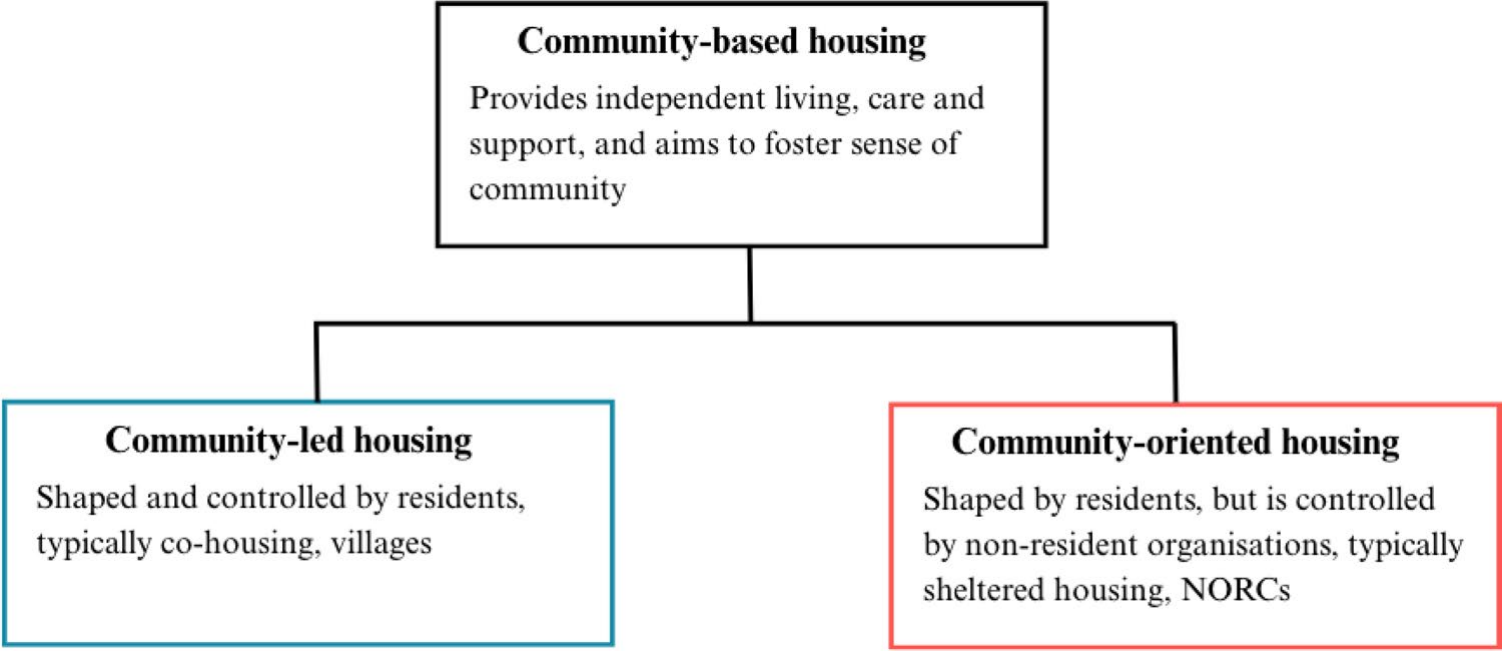
Different types of co-producing community-based housing—a conceptual distinction

We have shown that the co-production of community-based housing projects for older adults, across housing types and country contexts, can include various degrees of resident involvement. Initiatives differ in the way they provide dwellings for independent living (for example, some are purpose-built and designed by residents, others are develop within existing housing blocks; some rely on ownership as tenure, others on rental or mixed-tenure arrangements), care and support (whether formalised or informal, through neighbours or external personnel), and how they provide the environment for older adults to experience a sense of community (whether intentional communities or top-down organised, and whether smaller scale or larger scale). Using Czischke (2018) and other work on housing governance (e.g. Crabtree-Hayes 2024; Raco et al. 2023) to make sense of the previously described ways of resident involvement, we distinguish two types of community-based housing co-production: community-*led* and community-*oriented* co-production (see Fig. 1).

### Community-led housing

In housing studies, participation has long been presented as central to producing housing alternatives that meet residents’ needs, which is why a relatively large body of literature has researched community-*led* housing (Crabtree 2018). Community-led housing provision refers to housing where residents drive the development of housing, care and support, and a sense of community. Community-led housing can be defined as ‘housing shaped and controlled by a group representing the residents and/or the wider community that will be served by the housing’ (Heywood 2016, 8; see also Lang et al. 2020; Crabtree-Hayes 2024). Across Europe, community-led initiatives can also be found under the umbrella term ‘collaborative housing’ (Tummers 2016; Czischke 2018), encompassing different housing types with resident control and self-organisation at the centre.

Community-led housing is fundamentally *shaped* by its residents. This means projects are often initiated by prospective residents, meaning people who are considering moving into the development once it has been built. The architecture of the housing project is then designed to meet their preferences, and the community life is structured according to their decisions (Crabtree-Hayes 2024). Additionally, community-led housing is also *controlled* by its residents. This means that legally, the power to make choices lies with residents or representatives of residents (Crabtree-Hayes 2024). For example, the property can be owned by a legal entity governed by resident representatives (Beck 2020), as is often the case in co-housing and villages (see Fig. 1). This model of community control typically comes with some form of community ownership and community management (Jarvis 2015b). In the context of ageing, these standard definitions from housing studies must be expanded to encompass a focus on governance over support and care. In community-led projects, such as co-housing, residents influence their community environment and have significant control over how care and support are organised. This often involves residents managing care and supporting themselves, relying on interdependence and solidarity within the community (Puplampu 2020).

### Community-oriented housing

However, as discussed, many community-based housing projects for older adults are not entirely community-led. They can be top-down initiated by private and social housing providers, sometimes in collaboration with care providers or NGOs. Literature on co-production also discusses the increasing complexity of co-production in housing provision. Co-housing groups, for example, have been increasingly noted as working together with social housing systems and receiving subsidies from city governments (Droste 2015; Butzlaff et al. 2024; Czischke 2018; Riccò et al. 2024). Thus, on the one hand, self-organised housing groups include collaboration with different institutions, giving up control in certain areas of development. On the other hand, community elements and resident participation are integrated into top-down housing projects (Simmons and Birchall 2007; Schaff et al. 2023). This reflects a shift towards polycentric governance, which describes the often multi-directional relationship of co-production between government, organisations, and citizens (Ostrom 2010; 1990; Ostrom, Schroeder, and Wynne 1993) as well as trends towards more market-oriented housing provision in some countries (E.g. Raco et al. 2023).

To describe community-based housing that is not community-led, we introduce the term community-*oriented* housing, which refers to housing designed to facilitate a sense of community among residents. While the term is sometimes used in academic literature, it lacks a clear definition. These developments can be led by public housing providers, third-sector organisations, the private market, or through co-production between different stakeholders. For example, developments can include community rooms and other shared spaces designed and commissioned by a housing association with a resident community in mind (e.g. sheltered housing, Herbers and Meijering 2015). While the input of future residents must be considered in the design of the buildings, decisions regarding the development of the housing and care services are controlled by entities such as housing or care providers. Similarly, support and care services are organised formally, and the resident community is often determined by access criteria set by entities such as the social housing system of a city (E.g. Tinker et al. 2007). Thus, community-oriented housing for older adults is housing that is *shaped* by input from residents, but *not controlled* by its residents. These trends of letting residents participate in a top-down fashion are widely reflected in governance and participation literature (Saurugger 2010). For example, tenant participation in social housing has long been considered good practice in Europe (Simmons and Birchall 2007), with tenant associations and tenant management organisations taking over some responsibilities when providing housing services, for example, in the UK (ibid, page 574).

### Mixing community-*led* and community-*oriented* practices

**Fig. 2:**
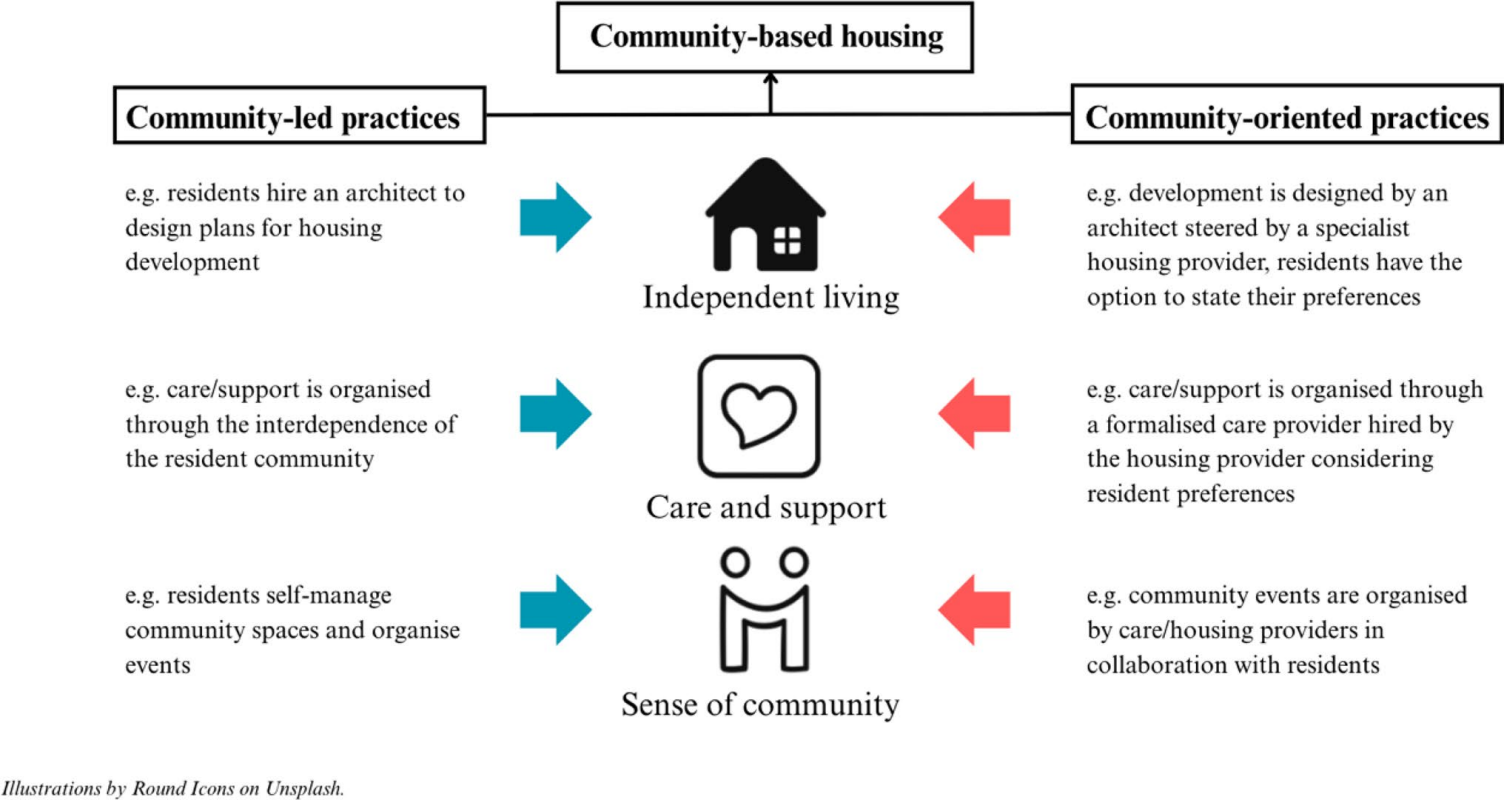
Conceptual framework: Resident involvement in community-based housing for older adults

Conceptually, the distinction between community-*led* and community-*oriented* housing appears straightforward (see Fig. 1). However, as the literature discussed in this paper shows, in practice, housing projects for older adults often combine elements of both. For example, co-housing projects for older adults can be initiated by NGOs or other organisations and are not always fully community-led. Similarly, NORCs can be resident-driven, sheltered housing can include resident involvement, and villages can be top-down and privately organised while delegating parts of the organisation to residents. Because classifying a community-based housing project as either community-led or community-oriented often falls short, we propose a focus on practices applied in these housing types. We understand *practices* as organised activities that shape and create independent living, care and support, and a sense of community in community-based housing. Instead of characterising one community-based housing project as community-oriented *or* community-led, we suggest viewing each project as a mix of both—comprising community-oriented and community-led practices (see Fig. 2).

We define *community-led practices* as practices that are shaped and controlled by (prospective) residents. Examples from the reviewed studies include residents initiating the development of independent living units (Graham, Scharlach, and Price Wolf 2014; Hou and Cao 2021, Pederson 2015), residents organising care through the interdependence and solidarity of the resident community (e.g. in co-housing, Puplampu 2020), or residents designing and self-managing community spaces and organising community events (Glass 2020; Glass and Norris 2023; Kavanagh et al. 2025). In contrast, *community-oriented practices* are shaped by the resident community but controlled by other stakeholders. This is the case, for example, when the built environment is designed by an architect, steered by a housing provider with resident interests or preferences in mind (Butler et al. 2021). Further, care and support can be organised through a formalised care provider hired by the housing provider after seriously considering resident preferences (e.g. Herbers and Meijering 2015). Similarly, community-building can be organised by care/housing providers in collaboration with residents through offered activities (Hou and Cao 2021), and flats can be allocated through the social housing system while considering resident preferences, instead of neighbours chosen by residents themselves (Stoisser and van Gent 2025).

**Fig. 3:**
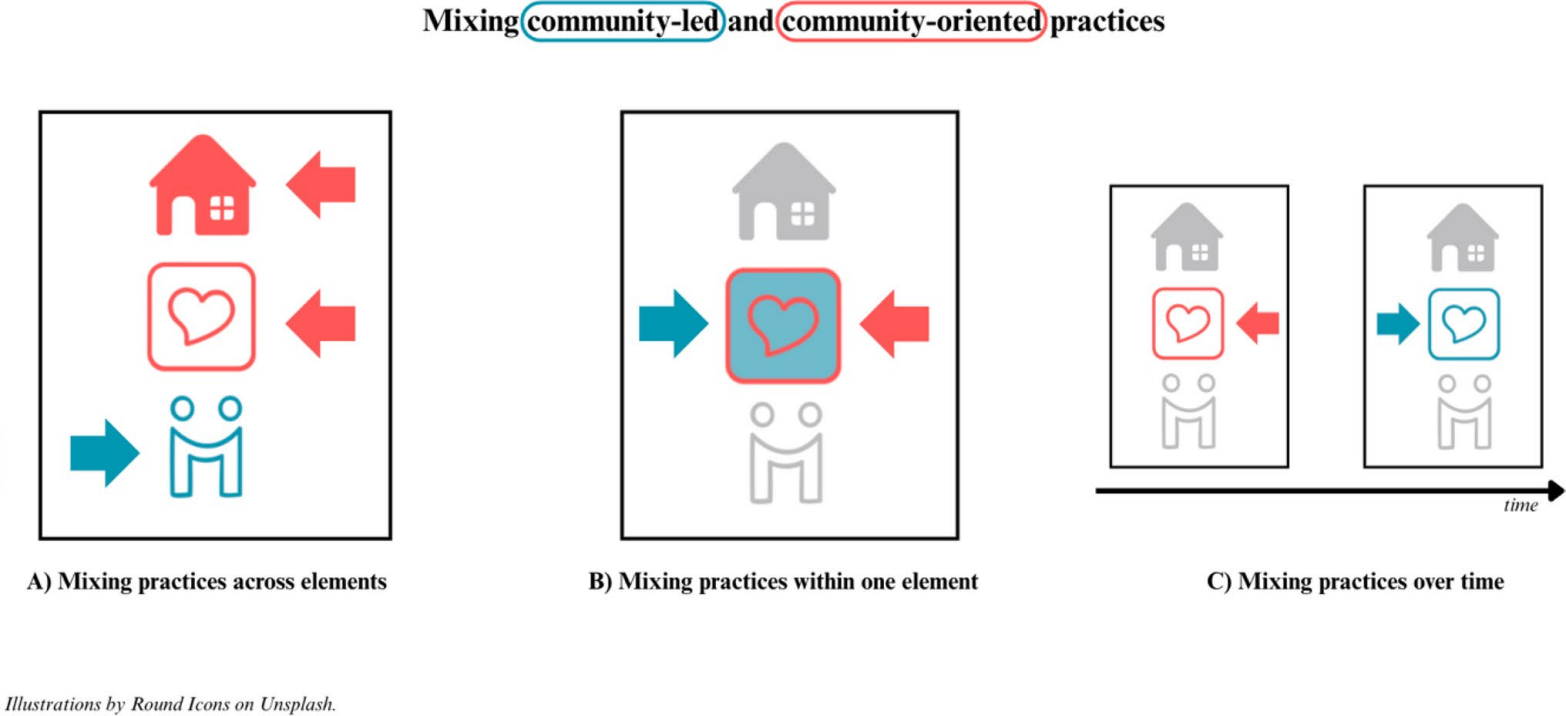
Mixing community-led and community-oriented practices in community-based housing

Many projects combine these two types of practices in different ways. Based on the literature reviewed, we identify three patterns, which we illustrate in Fig. 3 using the framework on community-based housing and resident involvement developed previously:

A. *Mixing practices across elements* A single housing initiative may adopt community-oriented approaches for one element (e.g. independent living), while relying on community-led practices for others (e.g. care or community-building). For example, in Lundman’s (2020) village model, the physical house (independent living) was developed by a property company with input from residents (community-oriented), while residents organised community events themselves (community-led).
B. *Mixing practices within a single element* One element, such as care and support, may involve both community-led and community-oriented practices simultaneously. For example, residents might coordinate informal support such as shared meals themselves, while external providers offer formal care services.
C. *Mixing practices over time* Some initiatives shift from community-oriented to community-led practices for one element over time. For instance, in the co-housing project studied by Smetcoren et al. (2022), care provision was initially organised through an NGO but later managed by the residents themselves. This enabled the project to be established more quickly while allowing for greater resident control as time passed.

Of course, the distinctions between community-oriented and community-led practices are conceptual, with blurry boundaries between the named practice types. The aim of this framework is to support an understanding of the different ways in which community-based housing can be co-produced. Focussing on practices allows to view community-based housing as an ongoing process, one that is managed, adapted, and lived in over time, rather than a fixed or static model. This perspective draws attention to how design, support, and governance evolve within developments.

These dynamics are shaped not only by the aspirations of residents, housing providers or project initiators, but also by structural conditions such as tenure arrangements and governance models. Ownership structures, in particular, play a key role in enabling or constraining resident control. Private or cooperative ownership, for example, may enable residents to exercise greater influence over decisions relating to the design, management, and everyday practices, while private or social rental models may impose more constraints—though this depends on local regulations.

This framework offers a first step towards recognising that top-down organised housing developments can and should incorporate meaningful opportunities for resident participation. At the same time, it challenges the common assumption in housing studies that housing alternatives must be resident-led. Rather, it emphasises that meaningful resident participation can take different forms and degrees, and that combining community-led and community-oriented practices may enhance inclusion, especially for those older adults who may not be able or willing to take on extensive roles and responsibilities. Crucially, however, some degree of resident control is essential for housing to be meaningfully co-produced. Without this, projects risk being community-based in name only. This framework should therefore not be used to justify less participation, but to prompt reflection on when and how older adults' voices are included in shaping housing futures.

## Discussion

Recognising the role of residents in the governance of community-based housing is crucial for academics and practitioners seeking to develop housing that supports autonomy, social connection, adequate care, and a sense of belonging in later life. Governance arrangements are not neutral backdrops; they influence how much control residents have over their living environments, who can participate, how support is organised, and whose needs are prioritised and met. Critically, governance practices often reflect and reproduce broader social inequalities linked to income, race and ethnicity, health, and care needs, affecting who can thrive and ‘belong’ in such housing settings (Cook et al. 2017; Greenfield et al. 2013; Davitt et al. 2017). As Stoisser et al. (2025) argue, governance arrangements are central to questions of *spatial justice—*that is, the fair distribution of resources, recognition, and decision-making power across different groups, shaping whose voices are heard, who can access community-based housing, and whose needs are prioritised in such housing models.

Building more inclusive and responsive forms of housing governance requires attention to how and when older people are involved in shaping their living environments. Encouraging community-led practices can help ensure that resident needs are met, that projects are developed in places where they are needed, and that older adults experience autonomy regarding the places that they live in. This not only applies to co-housing models, which are built on the premise of self-organisation, but also to more top-down arrangements. In the naturally occurring retirement community in the UK described earlier (Kavanagh et al. 2025), residents played a central role in initiating service development and co-designing a shared community space, despite the typically top-down nature of NORC governance. When supported sustainably, such resident involvement can enhance quality of life and contribute to more equitable and inclusive approaches to ageing.

However, fully self-organised housing models are not suitable for all older adults. As noted by others (Butzlaff et al. 2024), while community-led practices give residents a voice, they also place significant demands on their time, energy, and financial resources. In response, an increasing number of developments are adopting hybrid models that combine community-oriented and community-led practices. These models may offer a faster implementation process, greater social diversity, and lower individual burdens than traditional bottom-up housing alternatives (Jensen and Stender 2022). Villages that collaborate with other stakeholders, such as local governments, can also enhance affordability and sustainability (Graham et al. 2018). Thus, even when a full community-led organisation is not feasible, involving older residents in parts of the co-production process remains vital for developing more inclusive, responsive, and resident-driven housing solutions.

In housing studies, the term ‘housing alternatives’ typically refers to models that differ from market-based housing provision and conventional public housing provision (Tummers 2016; Czischke et al. 2020). Such initiatives are often characterised as self-managed, self-organised, or—as we have called it—community-*led*. However, when considering alternative housing for older adults, this definition is too narrow. Many older adults cannot, or do not wish to, remain in their long-term homes or move into institutional care. They may require affordable housing alongside care and support, but without the responsibility that full self-management entails. Addressing these needs calls for a broader understanding of alternative housing, one that includes community-*oriented* forms of housing provision that offer participation and support without assuming full resident control.

In combination, community-led and community-oriented practices offer a flexible framework for developing housing that enables older adults to *age in the right place*. Moreover, what is initially a community-oriented practice can evolve into a community-led practice by giving residents autonomy and power to manage their environments once they move in. This demonstrates that thinking about housing for older adults requires moving beyond a binary conceptualisation of either top-down-produced nursing homes *or* innovative bottom-up-developed co-housing groups. Using the approach suggested in this paper allows academics to describe the fluidity of governance arrangements and invites researchers and practitioners to consider how different configurations of community-led and -oriented practices influence the responsiveness of housing developments to the diverse needs and aspirations of older residents.

Finally, this article advocates for a broader understanding of ageing in place. Ageing in place is often defined as ageing in a private home where a person has lived most of their life. However, policy programmes built around this idea can lead to a lack of accessible care and housing options beyond this individual home (see Knijn and Hiah 2020, 172; Forsyth and Molinsky 2021). Interpreting ageing in place as ‘never moving’ risks neglecting the need for new housing solutions that might be crucial for some older adults. Community-based housing options can play an essential role in enabling ageing in the *right* place. By focussing on co-production practices across different dimensions of community-based housing (independent living, care and support, sense of community), this paper contributes to ongoing debates on how to develop more inclusive, diverse, and participatory housing alternatives to the prevailing binary of ageing at home versus ageing in institutional care.

### Limitations and further research

This paper has offered a conceptual exploration of the role of residents in community-based housing for older adults, highlighting the value of different degrees and forms of resident involvement. However, the quality of such housing projects, and whether they indeed promote a sense of community, ultimately depends on their implementation and on residents’ experiences. Of course, local and regional policy frameworks, as well as prevailing understandings of ageing and community, will significantly influence the way in which specific projects can develop. Moreover, concepts such as ‘participation’ and ‘co-production’ can be used to present a project as inclusive, but how and if residents can genuinely participate will depend on the intentions, practices, and capacities of the housing project and its stakeholders. There is a pressing need for empirical research that examines how community-*led* and community-*oriented* practices are organised and experienced in specific settings. Such studies could illuminate how residents and developers experience, interpret, and understand participation, and how they realise it in practice.

Further, governance is an inherently complex and multi-layered process. While the paper focussed on resident involvement in housing co-production, we acknowledge that this does not grasp the full complexity of housing governance. The roles and influence of public, non-profit, or market-based actors remain underexplored and warrant closer examination. Future research could expand our understanding of governance by incorporating these actors. Additionally, the paper builds mainly on literature in Northern/Western Europe and North America. While the general call for reflection on the role of residents in community-based housing can be applied to contexts universally, models of provision and policy frameworks differ significantly across contexts. There is a clear need for further empirical and conceptual work in diverse geographical contexts and sociopolitical settings.

## Conclusion

This paper addresses the role of residents in producing and governing community-based housing for older adults—a role that varies considerably across models and initiatives and remains conceptually underdeveloped in academic literature. Community-based housing offers potential benefits across three key dimensions: independent living, access to care and support, and a sense of community. Focussing on the role of residents in governing these dimensions, this paper proposes a conceptual framework that distinguishes between community-*led* and community-*oriented* practices. Community-*led* practices are those shaped and controlled by the (prospective) housing project residents, while community-*oriented* practices are influenced by residents but controlled by other actors. The paper acknowledges the diversity of emerging innovations in housing and care for older adults and invites practitioners and academics to reflect on the varied ways in which older residents can be meaningfully involved in housing governance.

## Data Availability

No datasets were generated or analysed during the current study.

## Footnotes

1 Institutional care settings are also called nursing homes or long-term care homes (see Howe, Jones, and Tilse 2013).
